# Screening Disinfection Byproducts in Arid-Coastal Wastewater: A Workflow Using GC×GC-TOFMS, Passive Sampling, and NMF Deconvolution Algorithm

**DOI:** 10.3390/jox14020033

**Published:** 2024-05-01

**Authors:** Muhammad Usman Siddiqui, Muhammad Sibtain, Farrukh Ahmad, Yasuyuki Zushi, Deedar Nabi

**Affiliations:** 1 Institute of Environmental Sciences and Engineering, School of Civil and Environmental Engineering, National University of Sciences and Technology, Islamabad 48000, Pakistan; 2 BioEnergy & Environmental Laboratory (BEEL), Masdar Institute Campus, Khalifa University, Abu Dhabi P.O. Box 127788, United Arab Emirates; 3 California Environmental Protection Agency, Cypress, CA 90630, USA; 4 National Institute of Advanced Industrial Science and Technology (AIST), Tsukuba 305-8569, Ibaraki, Japan; 5 GEOMAR Helmholtz Centre for Ocean Research Kiel, Wischhofstr. 1-3, 24148 Kiel, Germany

**Keywords:** disinfection byproducts, passive sampler, GC×GC-TOFMS, NMF spectral deconvolution, hazard assessment

## Abstract

Disinfection during tertiary municipal wastewater treatment is a necessary step to control the spread of pathogens; unfortunately, it also gives rise to numerous disinfection byproducts (DBPs), only a few of which are regulated because of the analytical challenges associated with the vast number of potential DBPs. This study utilized polydimethylsiloxane (PDMS) passive samplers, comprehensive two-dimensional gas chromatography (GC×GC) coupled with time-of-flight mass spectrometry (TOFMS), and non-negative matrix factorization (NMF) spectral deconvolution for suspect screening of DBPs in treated wastewater. PDMS samplers were deployed upstream and downstream of the chlorination unit in a municipal wastewater treatment plant located in Abu Dhabi, and their extracts were analyzed using GC×GC-TOFMS. A workflow incorporating a multi-tiered, eight-filter screening process was developed, which successfully enabled the reliable isolation of 22 candidate DBPs from thousands of peaks. The NMF spectral deconvolution improved the match factor score of unknown mass spectra to the reference mass spectra available in the NIST library by 17% and facilitated the identification of seven additional DBPs. The close match of the first-dimension retention index data and the GC×GC elution patterns of DBPs, both predicted using the Abraham solvation model, with their respective experimental counterparts—with the measured data available in the NIST WebBook and the GC×GC elution patterns being those observed for the candidate peaks—significantly enhanced the accuracy of peak assignment. Isotopic pattern analysis revealed a close correspondence for 11 DBPs with clearly visible isotopologues in reference spectra, thereby further strengthening the confidence in the peak assignment of these DBPs. Brominated analogues were prevalent among the detected DBPs, possibly due to seawater intrusion. The fate, behavior, persistence, and toxicity of tentatively identified DBPs were assessed using EPI Suite™ and the CompTox Chemicals Dashboard. This revealed their significant toxicity to aquatic organisms, including developmental, mutagenic, and endocrine-disrupting effects in certain DBPs. Some DBPs also showed activity in various CompTox bioassays, implicating them in adverse molecular pathways. Additionally, 11 DBPs demonstrated high environmental persistence and resistance to biodegradation. This combined approach offers a powerful tool for future research and environmental monitoring, enabling accurate identification and assessment of DBPs and their potential risks.

## 1. Introduction 

Water scarcity is a growing threat in South Asian and Middle Eastern countries, where access to clean water is limited and unevenly distributed, leading to social justice issues [1,2]. To address this, water reuse practices have gained attention as a sustainable solution [3]. Ensuring the safety of recycled treated wastewater is crucial to protect public health and the environment [4]. Pathogens in treated wastewater can pose significant health risks if not properly treated. Chlorination, a widely adopted method in municipal wastewater treatment, effectively targets pathogens, but also forms disinfection byproducts (DBPs) through reactions with residual organic matter [5]. Concerns about the ecological and human health effects of DBPs necessitate a comprehensive understanding of their occurrence and toxicity [6,7]. However, current regulatory guidelines and monitoring programs focus on only a limited number of DBPs, leaving a significant knowledge gap [7].

Innovative techniques like passive samplers can help bridge this gap in investigating DBPs. Passive sampling devices (PSDs) are popular due to their convenience, cost-effectiveness, and ability to capture a wide range of pollutants with lower detection limits [8,9]. They have been widely used in detecting organic chemicals in various environmental matrices, providing a more accurate assessment of exposure compared to conventional methods [10,11,12,13].

Various passive sampling devices (PSDs) have shown potential in monitoring organic pollutants in environmental waters, including PDMS, polyacrylate (PA), polyoxymethylene (POM), semipermeable membrane devices, low- and high-density polyethylenes, and ethylene–vinyl acetate [10,14,15,16,17,18]. PDMS or silicone passive samplers are cost-effective and suitable for a wide range of pollutants [14]. PSDs successfully monitor contaminants in treated wastewater, even in complex matrices [19,20]. They are also used for monitoring various DBPs in wastewater [21].

Passive samplers provide cleaner extracts, but their analysis using conventional GC-MS presents challenges in resolving co-eluting analytes and identifying nontarget pollutants [22,23]. Comprehensive two-dimensional gas chromatography (GC×GC) coupled with TOFMS is a promising technique to leverage the advantages of passive samplers [24]. GC×GC offers enhanced separation power compared to traditional GC, resolving complex mixtures [25]. Coupling GC×GC with TOFMS enables accurate mass measurements and compound identification [26,27]. GC×GC is valuable for separating halogenated complex mixtures and analyzing DBPs, predominantly halogenated compounds [28,29,30]. It facilitates the identification and quantification of a wide range of DBPs, contributing to understanding their occurrence and impacts [28,31]. Furthermore, the integration of peak alignment tools like GC Image [32], ChromaTOF Tile [32], and ChromCompare+ [33] plays a pivotal role in these non-targeted screening workflows. These tools automate the alignment and comparison of chromatographic data, significantly streamlining the process and reducing the traditional time and complexity involved in such analyses [34]. This advancement enhances the efficacy of discovering and identifying environmental pollutants, as demonstrated in recent studies [35,36].

Despite GC×GC-TOFMS’s advanced capabilities, real-world samples often have numerous unresolved peaks due to the full scan mode capturing all ions within a certain mass range, resulting in noise and coeluted components [37,38,39]. Spectral deconvolution, particularly using NMF-based methods, helps separate overlapping signals, facilitating more accurate identification of components in complex mixtures [40,41]. Zushi and coworkers [40] developed an NMF-based deconvolution method for GC×GC-TOFMS and evaluated its performance using a sediment sample. They found that increasing the mass resolution did not improve deconvolution, but a higher scan rate increased identifiable spectra. Deconvolution to high-resolution mass data successfully enhanced assignable spectra and the creation of an accurate mass-based spectral database even with selecting nominal mass in *m*/*z* precision for deconvolution processing, accelerating nontarget screening. Selecting an appropriate *m*/*z* precision setting for each mass resolution datapoint is crucial for noise reduction. Overall, NMF deconvolution in GC×GC-TOFMS accelerates nontarget and target screening in complex mixtures.

In this context of advancing analytical methods like spectral deconvolution, the framework established by Schymanski and coworkers [42] for structure classification has significance. This framework categorizes chemical structures based on evidence depth, from confirmed structures (level 1) to those identified by exact mass only (level 5). Level 2, termed probable structure, is particularly relevant to our study. It includes level 2a (library), where structures match the literature or library data unambiguously, and level 2b (diagnostic), where structures are identified based solely on experimental data. However, this classification could benefit from additional layers or subdivisions, especially in level 2a. Beyond library matches, incorporating factors like retention indices, expected GC×GC elution patterns, and physicochemical properties such as octanol–water partition coefficients could enhance the confidence level beyond the current scope of level 2a in Schymanski’s framework.

In addition to library matches, analyzing isotopic patterns, mass defects, and retention indices significantly boosts confidence in identifying halogenated compounds (HCs) in environmental samples. Utilizing isotopic signatures and mass defects of bromine and chlorine atoms improves the isolation and visualization of halogen fingerprints in HRMS data, which is crucial for non-targeted screening (NTS) with GC-HRMS systems [37,43,44,45] These techniques, combined with high chromatographic resolutions and scan rates, enhance the detection and identification of complex mixtures, including homologues and isomers. An innovative approach for calculating theoretical isotope patterns, which uses a novel tree-like structure for generating relevant isotopologues, has notably increased the accuracy of mass spectrometry data analysis. This method, available through the R-package enviPat and a user-friendly web interface, has proven its value in extensive molecular formula simulations, significantly advancing mass spectrometry [46]. Additionally, retention indices combined with mass spectral similarity scores are crucial for elucidating structures in environmental pollutants analyzed via gas chromatography-high-resolution mass spectrometry (GC-HRMS), effectively enhancing compound identification in complex samples [47,48,49,50].

In GC×GC analysis, methods described by [51,52] allow for the accurate calculation of retention indices for both the first and second dimensions. These indices, however, are primarily utilized for property predictions rather than directly enhancing confidence in peak assignments. When these indices are unavailable, the Abraham solvation model [53] can be employed. This model was used to create retention diagrams for 54 solutes across four stationary phase combinations, effectively predicting analyte positions in GC×GC chromatograms. Additionally, a recent GC×GC elution model [54] simplified the analysis of complex petroleum substances, correlating retention times with hydrocarbon classes and carbon numbers. However, the similarity of simulated and experimental GC×GC elution spaces has not been used in non-target analysis, indicating an opportunity for methodological improvements in non-target screening.

Several nontarget analyses of DBPs reported in the literature have been classified at a confidence level of two or lower. Lim and coworkers [55] reviewed such studies, focusing on DBPs resulting from ozonation in drinking water and wastewater. Another study [56] tentatively identified several hundred nitrogen-containing DBPs in chlorinated river water, conducted without the use of standards. In related research, studies [57,58] on ozonation and chlorination processes have identified compounds at a confidence level of two, suggesting probable structures based on analytical evidence. The ozonation study identified 127 brominated DBPs, including 15 new brominated carboxylic acids, while the chlorination study detected 20 and 11 chlorinated DBPs from tyrosine and tryptophan, respectively. Despite being classified as level two, these findings are crucial for understanding the formation of DBPs in real-world environmental water samples, and highlight the importance of DBPs in environmental chemistry and public health.

Wastewater effluent, containing a variety of DBPs, is often discharged into water bodies or used for landscaping and horticulture after chlorination [6,59]. Evaluating the identified DBPs in the effluent samples through (non)target screening is crucial for determining their classification as chemicals of concern [60]. This classification considers environmental properties to establish a hazard profile and assess potential implications for ecosystems and human health. Key environmental properties include Henry’s law constant (HLC), the octanol–water partition coefficient (log Kow), the organic carbon–water partition coefficient (log Koc), the bioconcentration factor (BCF), the biodegradation potential, and toxicity endpoints [61]. 

Research has linked DBPs to reproductive toxicity and developmental issues in fetuses [62,63]. Nitrogenous DBPs, such as haloacetamides, exhibit genotoxic and cytotoxic effects on both humans and zebrafish models [64]. Iodinated DBPs, such as iodo-trihalomethanes and nitrosamines, including NDMA, are notably cytotoxic and genotoxic [65]. Studies indicate that haloacetonitriles (HANs) and haloketones (HKs) are more toxic than trihalomethanes (THMs) [66], with toxicity increasing with the number of halogen atoms per molecule, highlighting greater hazards from brominated DBPs [67]. Additionally, 2,2-dichloroacetamide has been associated with significant health risks, including metabolic damage and cancer biomarkers in adult zebrafish [68]. DBPs identified through non-target analysis should be cross-referenced with comprehensive in vivo and in vitro toxicity databases available via the US EPA’s CompTox Chemicals Dashboard [69] to deepen understanding of their toxic profiles and mechanisms.

Considering all the aforementioned knowledge gaps, it is crucial to develop an effective approach for the analysis and evaluation of DBPs due to their widespread presence in treated wastewater. The objective of this study is to overcome these challenges by employing the following elements:

(a) PDMS passive samplers: These samplers provide cleaner extracts and lower detection limits compared to conventional sampling methods, enabling reduced matrix interference and more accurate DBP analysis.
(b) GC×GC-TOFMS: This advanced instrumentation offers enhanced separation power, facilitating better identification of DBPs compared to conventional GC-MS techniques.
(c) NMF deconvolution algorithm: Spectral overlap can occur even with advanced instrumentation. By utilizing the NMF deconvolution algorithm, overlapping peaks can be resolved and accuracy can be improved during compound identification, ensuring reliable analysis of DBPs.
(d) Stepwise exclusion of candidate structures: This approach utilizes a set of criteria to minimize misassignments of peaks and narrow down the candidate list of DBPs, ensuring more reliable analysis results.
(e) Rapid hazard assessment: By incorporating tools like EPI Suite^TM^ and CompTox Chemicals Dashboard, potential risks associated with identified DBPs can be quickly understood, providing insights into their potential environmental impacts.

By integrating these approaches, our study aims to provide a robust analysis of DBPs in wastewater, enabling better understanding of their presence, characteristics, and potential risks. This research is essential for the development of effective wastewater treatment strategies and for the protection of public health and the environment.

## 2. Materials and Method 

### 2.1. Passive Sampler Preparation

AlteSil™ Silicone rubber sheets, purchased from Altec Products, Ltd. in Cornwall, UK, were used in this study. These translucent sheets were made from food-grade silicone and had dimensions of 600 mm × 600 mm with a thickness of 3 mm and a hardness of 60 Shore. To prepare the rubber strips for experimentation, the silicone rubber sheets were cut into 21.5 cm × 16 cm strips, each weighing 3.77 g. The strips underwent a sequential washing process. First, they were soaked in Milli-Q water in an incubator shaker at 200 rpm for 24 h. Then, they were immersed in a mixture of acetone and hexane (1:1) and shaken at 200 rpm for another 24 h. Afterward, the strips were removed from the solution and immersed in methanol in an incubator shaker at 200 rpm for an additional 24 h. Finally, the strips were rinsed and stored in airtight glass bottles containing Milli-Q water until they were deployed in the wastewater treatment plant. For the experimental procedures, HPLC grade solvents (hexane, acetone, and methanol) purchased from Sigma Aldrich were used. Throughout the experiments, Milli-Q water with a resistance of 18.2 MΩ·cm was used to ensure water purity.

### 2.2. Field Deployment of Passive Samplers 

To secure deployment of the passive sampler strips, stainless steel cages were utilized, and stainless-steel paper clips were used for anchoring strips to the inside of the cage. For added protection against fast-moving particles in the wastewater, the cages were wrapped with three layers of aluminum mesh. These assembled cages were then attached to stainless steel rods. To ensure a precise evaluation of the formation of DBPs, we strategically positioned the passive samplers in duplicates at two key locations within the municipal wastewater treatment plant. The first was placed at the inlet, prior to the chlorination step, and the second at the outlet, immediately after the chlorination step. Here, the samplers at the pre-chlorination stage effectively served as a field control, capturing conditions without chlorination. This setup allowed us to effectively compare the samples from the pre-chlorination and post-chlorination stages, thereby providing a clear understanding of the formation of DBPs as a result of the chlorination process.

As a control measure, one of the passive sampling strips was reserved as a field blank, which underwent identical exposure conditions as the deployed strips, except that it was not immersed in the wastewater. The purpose of the blank strip was to assess laboratory or background interference levels, which need to be subtracted from the detected compounds during analysis.

The silicone strips remained deployed for 30 days at the Al Wathba-2 Municipal Sewage Treatment Plant, located in Abu Dhabi, United Arab Emirates. This plant has a maximum treatment capacity of 300,000 m^3^/day (total treatment capacity of 430,000 cubic meters per day), to accommodate a population of over 1,500,000 units.

### 2.3. Retrieval and Extraction of Passive Samplers

Passive samplers were retrieved from the wastewater treatment plant and transported to our laboratory at Masdar Institute in Abu Dhabi. Each of the passive sampler strips, including those deployed at both the pre-chlorination and post-chlorination stages, was independently processed to produce separate extracts. Specifically, four strips (two from each stage) resulted in four distinct extracts for analysis, aimed at comparing the differences in DBP formation due to the chlorination process. Additionally, two field blank strips were also processed in the same manner, yielding two separate extracts. Therefore, a total of six extracts (four from the deployed samplers and two from the field blanks) were analyzed to ensure a comprehensive evaluation of DBPs and to control for any potential background or laboratory contamination.

For extraction, the strips were gently rinsed with Milli-Q water and wiped to remove the biofilm from their surfaces. They were then rinsed again with Milli-Q water. The strips underwent three extractions using 20 mL of a 1:1 (*v*:*v*) acetonitrile/methanol mixture. The extractions were performed by shaking the strips overnight at 200 rpm. The resulting extracts from the three extractions were combined and reduced to a volume of 1 mL using a rotary evaporator. In our non-target screening study, specific extraction recovery experiments on AlteSil™ silicone rubber sheets were not conducted, given that this approach primarily focuses on the broad analysis of unknown compounds, obviating the need for individual compound recovery tests. The literature indicating high recovery rates for analogous analytes on these sheets—approximately 70% for organophosphate flame retardants [70], and nearly complete for hydrophobic chemicals such as PCBs, PAHs, and chlorobenzenes [71]—sufficiently validates the efficacy of the extraction method employed in our study. Subsequently, the passive sampler extracts were transported to the National Institute for Environmental Studies in Tsukuba, Japan for further analysis using GC×GC TOF-MS.

### 2.4. GC×GC TOFMS Analysis 

The passive sampler extracts were analyzed using GC×GC-TOFMS. The MS instrument used was the JMS-T100GCV 4G-N TOF-MS (JEOL, Tokyo, Japan), and the modulator employed was the KT2006 GC×GC system (Zoex, Houston, TX, USA). The first GC column utilized was the Restek RXI 1MS/Sil (30 m × 0.25 mm i.d., 0.25 μm film thickness, GL Sciences, Tokyo, Japan), and the second column was the SGE BPX-50 (1 m × 0.1 mm i.d., 0.1 μm film thickness, Sigma-Aldrich, St. Louis, MO, USA).

For injection, a volume of 1 μL was used at a pressure of 317.3 kPa and a temperature of 70 °C in splitless mode, with helium as the carrier gas. The GC oven temperature was initially set at 70 °C for 2 min and then ramped up to 300 °C at a rate of 5 °C/min, followed by a 10 min hold at 300 °C. The modulation period was set at 6.5 s. The TOFMS detector operated at an ionization voltage of 70 eV for electron impact, with the microchannel plate detector voltage set to 2300 V. The mass range scanned was from 33 to 800 *m*/*z*, with a scan rate of 50 Hz. These instrumental settings were adapted from previous studies on the analysis of halogenated compounds, ensuring a comprehensive screening of DBPs [27,37,38]. Evaluation of the acquisition frequency in previous work [40] demonstrated optimal spectral quality at 50 Hz. However, the impact of varying the mass range was not specifically evaluated in this study, presenting a potential direction for future research.

### 2.5. NMF Deconvolution

The NMF deconvolution was performed for GC×GC-TOFMS data (MS resolution 8000 with scan rate, 50 Hz) using R version 4.1.2 with the R code (GC×GC-NMF-Classification_v1.1.3) developed by Zushi and coworkers [40]. Peak selection was accomplished using an inverse watershed algorithm [72] in GC Image version 2021 R-1, following the general recommendations in the GC Image user guidelines. For the selected peaks, NMF was applied using the non-negative double singular value decomposition (NNSVD) seeding method. The Frobenius algorithm was chosen as the baseline NMF algorithm, and the NNSVD method, which generated initial matrices, was used for spectral deconvolution. These settings effectively reduced computational costs [40]. Considering the exceptional separating ability of GC×GC, it was assumed that there were at most four overlapping peaks, and the deconvoluted spectra were organized into a new chromatogram called a layer. Four deconvoluted layers were analyzed in this study. The subsequent analysis involved using GC Image and performing a NIST Library search for 350,643 spectra with the main library to identify compounds corresponding to the deconvoluted peaks. The retention times (RTs) for the GC1 and GC2 columns of the peaks were linked and stored in the peak list. The same procedure was also applied to the original chromatogram.

### 2.6. Sequential Filtering Process for Accurate Identification of Disinfection Byproducts

**Figure 1:**
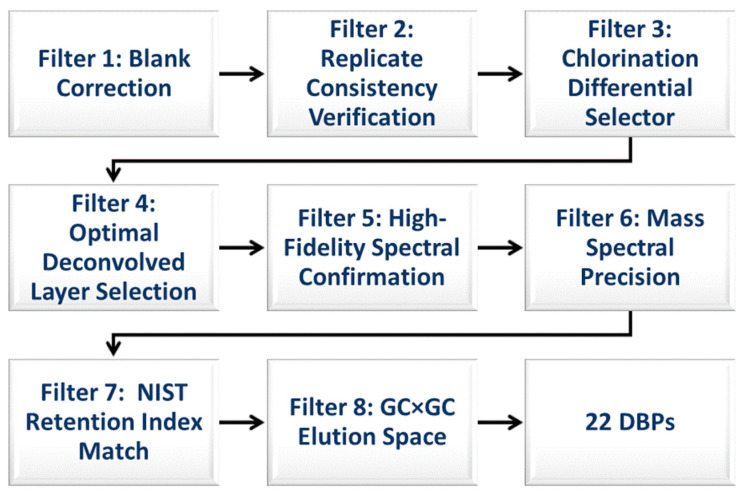
Sequential filtering workflow for DBP identification. Filter-1 eliminates false positives by removing peaks present in field blanks. Filter-2 assesses peak reproducibility across replicates, and Filter-3 selects chlorination-related peaks. Filter-4 chooses the peak with the highest match factor from deconvolved data. Filter-5 confirms peak fidelity against spectral libraries. Filter-6 utilizes isotopic pattern similarity and mass accuracy for peak precision, Filter-7 compares experimental retention index data from the NIST Chemistry WebBook with calculated data for DB-1, and Filter-8 applies two-dimensional chromatographic modeling to validate the position of the DBPs GC×GC chromatogram. The process ensures robust identification by systematically minimizing false positives and validating true positives.

The obtained data underwent a stepwise procedure for screening using the following set of criteria (Figure 1).

**Filter-1: Blank Correction—**This first step involved removing identified peaks present in the field blank sampler from the list of peaks obtained from the field samplers. This elimination of peaks detected in blank samplers helped eliminate false positives, ensuring a more accurate representation of the real compounds present in the effluent. 

**Filter-2: Replicate Consistency Verification—**In the second step, only peaks detected in both replicate samplers were considered, while peaks exclusive to one sampler were disregarded. This filtering process enabled differentiation between true compounds and potential artifacts or inconsistencies in the sampling process, thus assessing the reproducibility of the sampling method. 

**Filter-3: Chlorination Differential Selection—**For the third step, only peaks that emerged in the samplers deployed after the chlorination step, but were absent in the pre-chlorination samplers, were considered representative of disinfection byproducts. 

**Filter-4: Optimal Peak Selection—**If common peaks were detected in multiple layers obtained after NMF deconvolution, only the peak with the highest match factor score was selected, while others were disregarded. This step ensured the retention of peaks consistently present across real samples, reducing false positives originating from noise or random variations.

**Filter-5: High-Fidelity Spectral Confirmation—**It aimed to identify and exclude peaks with poor matches (i.e., <800) to the available spectra in the in the NIST Library. By considering the similarity of peaks to the reference spectra, this filter enhanced the accuracy and reliability of peak identifications.

**Filter-6: Mass Spectral Precision—**To further enhance the confidence in peak assignments for DBPs, we conducted mass spectral similarity and mass accuracy calculations. Utilizing the enviPat web interface [73], we calculated the theoretical isotope patterns, including isotopologue distributions [46], and compared these with observed isotopic patterns from mass spectrometry data. The application of cosine similarity in this context, ranging from 1 for a perfect match to 0 for no correlation, significantly bolstered our confidence in correctly identifying the DBPs. Additionally, by comparing the theoretical and measured monoisotopic masses of DBPs, we ensured precision in our identifications, further reinforcing the reliability of our peak assignment process.

**Filter 7: NIST Retention Index Match—**In the NIST Chemistry WebBook, SRD 69 [74], experimental retention index data for 11 DBPs on nonpolar columns were available. These data were compared with the retention index data calculated for DB-1 (poly(dimethylsiloxane)), which we used as a first-dimension column in our study. The retention index for DB-1 was calculated using Abraham solute descriptors, as described in [53] (Tables S1–S3). A close match between the experimental and calculated retention index data helps in confirming the accuracy of our peak assignment.

**Filter 8: GC×GC Elution Space—**To apply this filter, we utilized the methodologies from [53,75] to predict the GC×GC elution diagram for DBPs. We selected stationary phases similar to those in our study, specifically DB-1 (poly(dimethylsiloxane)) and HP-50+ (poly(dimethyldiphenylsiloxane) with 50% diphenylsiloxane), as described in Seeley et al. (2009). The Abraham solute parameters (ASPs) for DBPs were obtained from the UFZ-LSER database [76], with estimated ASPs for some DBPs due to limited experimental data. These ASPs, alongside the system coefficients for DB-1 and HP-50, were used to calculate retention indices and predict the GC×GC elution diagram for the DB-1× HP-50 column combination. The Procrustes disparity and correlation coordination scores [77] were utilized to assess the alignment between predicted and experimental GC×GC elution diagrams of DBPs. The Procrustes score indicates spatial similarity, with lower values denoting greater alignment. Correlation coordination scores reflect the degree of correlation, where absolute values close to 1 suggest a strong correlation, and those near 0 indicate no correlation. Together, these metrics provide a robust assessment of the concordance between the model predictions and the actual chromatographic behavior of DBPs.

By sequentially applying these filters, our procedure effectively screened the data while adhering to specific scientific criteria for each filter. This systematic approach identified peaks consistently present across real samples, emphasizing their potential significance, while minimizing false positives arising from noise or random variations.

### 2.7. Hazard Screening Analysis

The fate, behavior, and toxicity assessments of the identified disinfection byproducts were conducted using EPI Suite^TM^ 4.1 software. The physicochemical properties, including HLC, log Kow, and log Koc were estimated using the HENRYWIN™, KOWWIN™, and KOCWIN™ modules of EPI Suite^TM^. Additionally, the biodegradation potential and the toxicity of the DBPs were evaluated using the BIOWIN™ and ECOSAR™ modules of EPI Suite^TM^. The potential activities of DBPs relating to developmental toxicity, mutagenicity, and endocrine disruption were assessed using the CompTox Chemicals Dashboard v2.2.1. Furthermore, the bioassays database within the CompTox Chemicals Dashboard v2.2.1 was searched to identify the number of bioassays in which the DBPs were tested and found to be active. Comparisons were made between the predicted properties and toxicity endpoints and the relevant regulatory guidelines and standards in order to determine the potential implications of the DBPs.

## 3. Results and Discussion

### 3.1. Synergy of Passive Samplers and GC×GC

**Figure 2:**
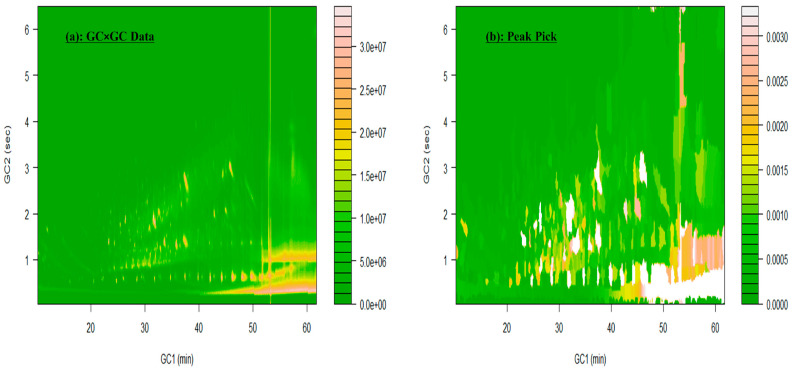
Peak picking and spectral deconvolution using an inverse watershed algorithm for NMF analysis, with retention time positions (RT1 for GC Column 1 and RT2 for GC Column 2) and mass spectra for NIST library search. (**a**) Raw GC×GC-TOF-MS Chromatogram of post-chlorination sample prior to spectral deconvolution. (**b**) Peak picking result obtained through the inverse watershed algorithm.

The GC×GC chromatogram displayed in Figure 2a showcases the enhanced separation capabilities of this analytical technique. The chromatogram exhibits well-resolved peaks, with several hundred compounds clearly separated. The separation achieved by GC×GC allows for the detection and characterization of a larger number of compounds which otherwise would have been challenging to resolve using a single GC column.

Furthermore, the raw GC×GC chromatogram demonstrates a remarkably clean appearance with minimal matrix effects, as depicted in Figure 2a. This outcome was anticipated, given that PDMS passive sampler extracts utilized in this study are known to provide cleaner extracts compared to normal solvent extracts of effluent samples. The reduced matrix effects in the chromatogram enhance the reliability and accuracy of compound detection and analysis.

The inverse watershed algorithm was employed for peak picking in the GC×GC chromatogram, as shown in Figure 2b. This algorithm successfully identified and marked several hundreds of peaks, indicating the presence of numerous compounds in the sample. The effective peak picking process plays a crucial role in subsequent analysis, such as spectral deconvolution and compound identification.

The results obtained from peak picking using the inverse watershed algorithm validated its performance in accurately detecting and selecting peaks of interest in the GC×GC chromatogram. By identifying and localizing these peaks, the algorithm enabled further analysis and facilitated the subsequent steps of spectral deconvolution and compound identification.

Overall, the combination of passive samplers, GC×GC, and the use of the inverse watershed algorithm for peak picking provided valuable insights into the separation and detection capabilities of this analytical technique. This methodology offers a reliable and effective approach for the analysis of complex mixtures, enabling comprehensive compound characterization and identification.

### 3.2. Enhancing the Identification of Disinfection Byproducts through NMF Deconvolution

**Figure 3:**
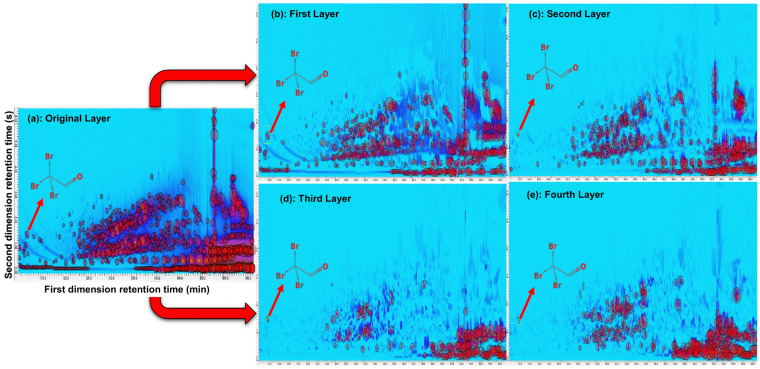
The process of baseline correction and peak detection using GC Image software Version 2020 for GC×GC-TOFMS chromatogram. Panel (**a**) displays the original GC×GC-TOF-MS data after baseline correction, but before spectral deconvolution. Subsequently, (**b**–**e**) represent the progressive stages of spectral deconvolution in Layer 1, Layer 2, Layer 3, and Layer 4, respectively. The highlighted blob in the figure tentatively corresponds to acetaldehyde tribromo, a potential identification.

Figure 3 illustrates the process of baseline correction and peak detection using GC Image software version 2020 for GC×GC-TOF-MS analysis. This software is a valuable tool for enhancing the accuracy and reliability of DBP identification in complex samples. Figure 3 also depicts the various stages of spectral deconvolution, emphasizing their impact on peak detection and identification.

The first stage, as depicted in Figure 3a, involves baseline correction, which effectively removes noise and improves data quality. Subsequently, the spectral deconvolution process occurs in multiple layers. Figure 3b showcases Layer 1, where false peaks are eliminated, revealing new peaks that contribute to more precise compound identification. Moving to Figure 3c, Layer 2 demonstrates further reduction of background noise, enhancing the detection of additional compounds and improving sensitivity for a comprehensive DBP analysis. Figure 3d represents Layer 3, where the spectral deconvolution process reduces the number of overlapping peaks and potential artifacts by eliminating multiple hits in close proximity. This step ensures more accurate DBP identification. Lastly, Figure 3e displays Layer 4, showing the lowest intensity of peaks after the deconvolution process. This final stage refines peak intensity and provides a clearer representation of the detected compounds.

**Figure 4:**
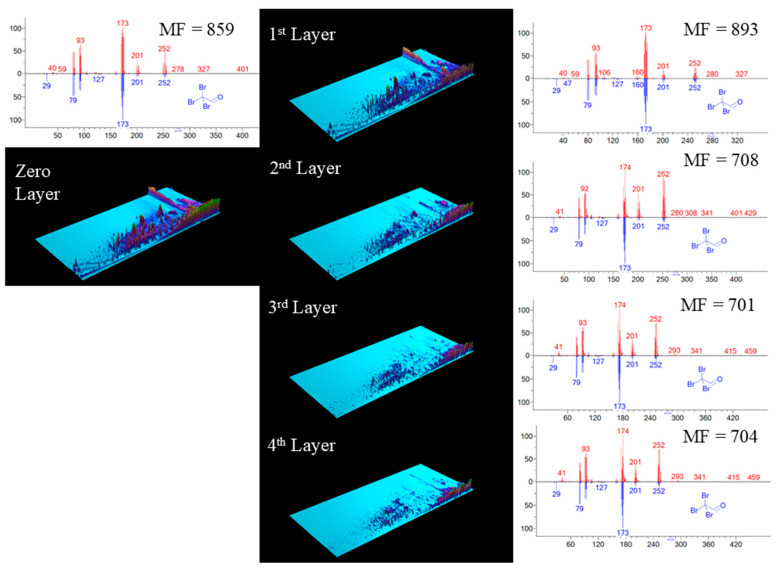
Performance of NMF spectral deconvolution in DBPs analysis. The zero layer presents raw data, subsequently deconvolved into four layers using non-negative matrix factorization (NMF). Illustrated is the mass spectrum of tribromoacetaldehyde, compared between the NIST reference library (blue) and the sample (red) in a head-to-tail alignment for each layer. The match factor (MF) for each layer is indicated, illustrating the enhanced accuracy of the NMF method in spectral analysis.

The power of NMF deconvolution is illustrated with a select peak corresponding to the selected disinfection byproduct, acetaldehyde tribromo, in each deconvoluted layer in Figure 4. The match factor increased significantly to 893 in the first layer, indicating a strong similarity between the obtained spectrum and the reference spectrum. The match factor of this peak in the raw chromatogram was 859. However, as the layers became deeper, the match factor decreased to 708, 701, and 704 in the second, third, and fourth layers, respectively. This decrease can be attributed to the presence of residual spectra in the deeper layers after the extraction of pure spectrum in the primary layer [40]. Despite the decrease in the match factor, the deep layers still contained spectra with potential recognition, suggesting the presence of additional compounds or variations in the analyzed sample.

Taken together, the use of NMF in the deconvolution technique significantly improved the match factor of identified peaks, with an average increase of 17.12%. This enhancement indicates a better alignment between obtained spectra and reference spectra, boosting confidence in compound identification and enhancing analysis reliability. Moreover, the spectral deconvolution process unveiled the presence of seven disinfection byproducts that were initially overlooked in the raw chromatogram (Table 1). This finding emphasizes the deconvolution technique’s ability to uncover additional peaks that may have been masked or missed during the initial analysis. These previously unidentified disinfection byproducts hold potential implications for water quality assessment and monitoring. However, it is essential to consider the noise and potential accumulation of trivial information in deeper layers, emphasizing the need for the rapid automated screening of layers to ensure efficient and reliable analysis.

### 3.3. Implementation of Sequential Filtering Process for Accurate Identification of Disinfection Byproducts

The performance of the spectral deconvolution technique was further enhanced by implementing a series of data filters, as illustrated in Figure 1, to improve the accuracy and reliability of peak assignment to disinfection byproducts. These filters systematically narrowed down the number of peaks to focus specifically on the target compounds.

The first filter involved blank correction, reducing the initial 7158 peaks to 3919. The second filter, which accounted for replicate correction, eliminated 782 additional peaks attributed to external contamination or false peaks. The third filter was designed to select peaks exclusive to the post-chlorination step, thereby identifying potential disinfection byproducts. This filter resulted in the removal of 1117 peaks.

To ensure the selection of only the most representative peaks, the fourth filter was implemented to choose a single peak based on the highest match factor score among identical peaks in multiple layers of the same chromatogram obtained after deconvolution. Consequently, 1350 peaks were excluded.

The final filter aimed to select only peaks with a match factor above 800, resulting in the identification of 22 potential disinfection byproducts. These peaks are summarized in Table 1. The match factor values for these peaks ranged from 933 to 810, indicating excellent to good matches with the NIST library. Among the 22 tentatively identified DBPs, 11 were commonly known DBPs, while 11 had not been previously reported as DBPs in the literature, to the best of our knowledge. Notably, 7 out of the 22 DBPs were initially undetected in the raw chromatogram due to spectral peak overlapping. The application of NMF spectral deconvolution enabled their identification, and the corresponding match scores for each layer can be found in Table 1. These rigorous filtering criteria greatly enhanced the confidence and reliability in the identification of the target compounds.

To further ascertain the accuracy of peak assignments in our analysis, Table 2 presents the molecular ion peak observations, corresponding isotopic pattern and mass error data for the 22 DBPs. The molecular ion peak, crucial for the calculation of isotopic patterns and mass errors, was detected in the reference (NIST) spectra of 14 DBPs (IDs: 1, 2, 3, 5, 7, 8, 9, 10, 14, 15, 17, 20, 21, and 22). Excluding DBP ID 7, which scored 0.75, possibly due to spectral interference of a dehydrogenated ion, the cosine similarity scores for these DBPs exhibited an excellent match, ranging between 0.994 and 1. The mass errors for this group were observed to be within 66.9–80.8 ppm.

Conversely, the molecular peaks were notably faint in the reference spectra for four DBPs (IDs: 13, 16, 18, and 19). Specifically, for DBP ID 13, the molecular peak’s low intensity precluded the calculation of the isotopic pattern and mass error. The cosine similarity scores for DBP IDs 18 and 19 indicated moderate similarity (0.402 and 0.519, respectively), except for DBP ID 13, which achieved an excellent match of 0.995. The mass errors for these DBPs fell within the range of 70–100 ppm.

The absence of molecular ion peaks in the reference spectra for four DBPs (IDs: 4, 6, 11, and 12) hindered the calculation of cosine similarity scores. Overall, where molecular peaks were pronounced in the reference spectra, isotopic pattern similarity was found to be excellent, indicating a robust correlation between observed and theoretical isotopic patterns for most DBPs. This result enhances confidence in peak assignments. The mass error values, reflecting the deviation between theoretical and measured monoisotopic masses, remained within a reasonable range for mass spectrometry analyses, averaging around 75.9 ppm, in line with the TOFMS’s resolution of 8000.

The experimental retention index data for nonpolar columns from the NIST Chemistry WebBook were compared to calculated data for our first-dimension column, using Abraham solute descriptors (Table 2 and Table S3). Excluding DBP 13, identified as an outlier, the root mean square error (RMSE) for the remaining 10 data points was found to be 28. The reported retention index for DBP 13 differed significantly, as it was an isothermal Kovats Index on a packed nonpolar column, unlike the other 10 DBPs with temperature programmable retention index data on a capillary column. This disparity accounts for the substantial deviation observed for DBP 13. In conclusion, the RMSE affirmed the agreement between calculated and NIST Chemistry WebBook measurements, enhancing peak assignment accuracy and confidence.

**Figure 5:**
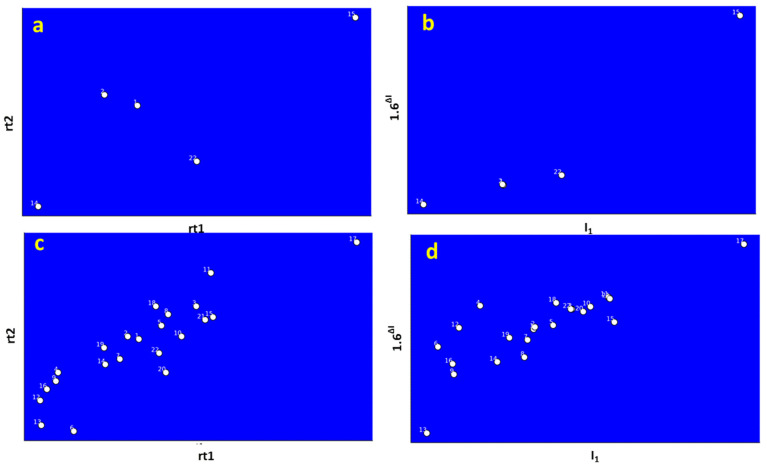
Comparison of observed and predicted GC×GC elution patterns for DBPs. The upper panels (**a**,**b**) display the observed and predicted elution patterns for five DBPs, derived using experimental Abraham solute descriptors. The lower panels (**c**,**d**) expand the comparison to all 22 DBPs, using estimated descriptors. Here, rt1 and rt2 represent the observed retention times in the first and second dimensions, respectively. Correspondingly, I_1_ and 1.6^ΔI^ denote the predicted first dimension retention index and modified second dimension retention parameter, with ΔI = I_2_ − I_1_, where I_2_ is the second-dimension retention index. Detailed elution order information for these compounds is available in Table S3 of the supplementary material.

To further ascertain the identities of DBPs, our study extended beyond match factor, spectral similarity, and mass error calculations to compare the elution positions of DBPs in both predicted and observed GC×GC elution spaces, as depicted in Figure 5. This comparison serves as an additional validation step, with the congruence of the predicted and observed GC×GC elution spaces providing confirmation of accurate peak assignments.

Figure 5a,b show the observed and predicted GC×GC elution patterns for five DBPs. These five DBPs were selected due to the availability of experimental Abraham solute descriptors, essential for accurately predicting the first- and second-dimension retention indices needed to construct the GC×GC elution space. In contrast, Figure 5c,d present the observed and predicted elution patterns for all 22 DBPs. In this case, the first- and second-dimension retention indices were calculated using estimated Abraham solute descriptors due to the absence of experimental values, impacting the construction of the GC×GC elution space. 

The Procrustes disparity between Figure 5a,b was notably low at 0.0285, signifying a high degree of similarity between the predicted and experimental GC×GC elution spaces for the five DBPs. For the larger dataset of 22 DBPs, as shown in Figure 3c,d, the Procrustes Disparity was higher at approximately 0.0793, but still indicated a reasonable degree of similarity.

Correlation analysis further elucidated these observations. A remarkably strong positive correlation, with a coefficient of approximately 0.993, was found between the predicted first dimension retention index (I1) values and the experimental first dimension retention time (rt1) values for the five DBPs. This high correlation along the first dimension highlights the accuracy of the predicted elution patterns. For the 22 DBP dataset, a very strong positive correlation of approximately 0.975 was observed between the predicted I1 values and the experimental rt1 values, reaffirming the predictive model’s effectiveness.

However, the correlation along the second dimension was slightly lower. For the five DBPs, the correlation between the predicted second dimension retention parameter (1.6^ΔI^) values and the experimental second dimension retention time (rt2) values was strong at approximately 0.820. For the 22 DBPs, this correlation was moderately strong at approximately 0.726. The diminished predictive performance in the second dimension compared to the first can be attributed to the use of a coefficient value of 1.6 in the second-dimension retention parameter (1.6^ΔI^), which may vary from 1 to 2 for better prediction of second dimension times. This aspect was not explored in our study, potentially explaining the lower similarity values along the second dimension.

The lower similarity in the larger dataset can be attributed to the reliance on estimated Abraham solute descriptors for calculating retention indices. These estimations are generally less accurate than experimental values, as indicated by considerable estimation errors in certain DBPs (detailed in Table S1 of the Supporting Information). This discrepancy likely contributed to greater dissimilarity between the observed and predicted GC×GC elution spaces, especially when compared to the dataset with solely experimental descriptors.

Taken together, the alignment between the predicted and experimental GC×GC elution spaces, particularly along the first dimension, corroborates the effectiveness of our methodological approach in increasing confidence in the peak assignments of DBPs. This finding is in harmony with the earlier mass spectral similarity results, collectively reinforcing the robustness and reliability of our analytical strategies in DBP identification.

**Figure 6:**
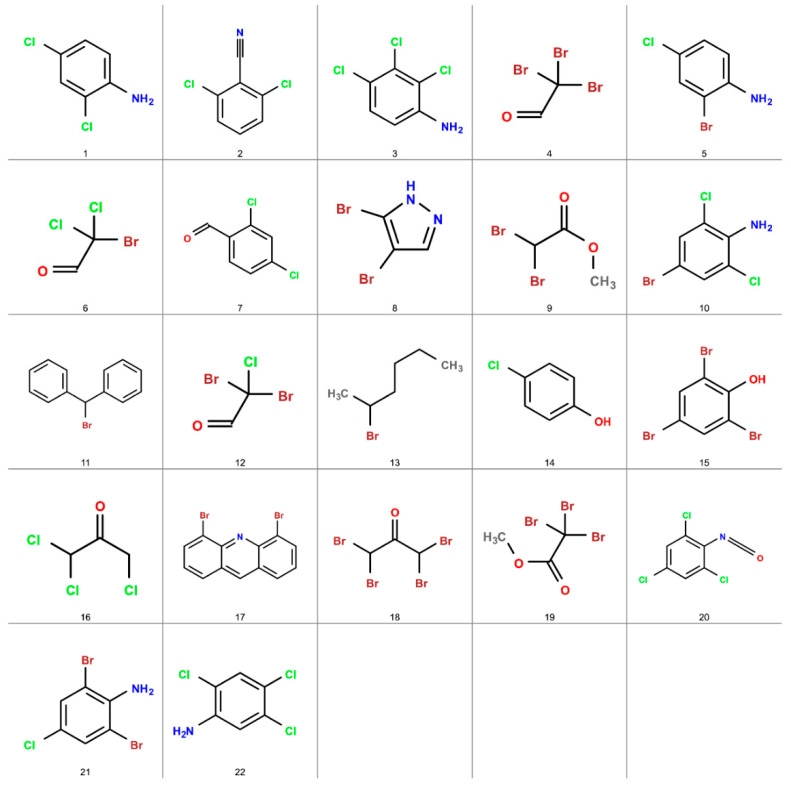
Structural representations of the 22 tentatively identified disinfection byproducts.

The prevalence of brominated DBPs in the detected samples (Figure 6) can be attributed to the susceptibility of coastal treatment plants, such as Al Wathaba Wastewater Treatment Plant in this study, to seawater intrusion. As supported by Osman et al. [78], there is evidence of both hypersaline groundwater leakage into sewage lines and seawater intrusion at the Abu Dhabi coast. Furthermore, Chowdhury [79] demonstrated that seawater intrusion can lead to the formation of brominated and iodated DBPs, confirming the potential contribution of such intrusions to the presence of brominated DBPs. Additionally, Ged et al. [80] quantitatively assessed the formation of brominated DBPs during chlorination in the context of seawater intrusion, highlighting the significant impact of this intrusion on the elevated concentrations of brominated DBPs. These collective studies offer valuable insights into the possible sources of brominated DBPs and underscore the influence of both hypersaline groundwater and seawater intrusion in their formation.

### 3.4. Hazard Profiling of Detected Wastewater DBPs

Evaluating the hazards associated with contaminants in effluent is crucial for safe wastewater reuse. We performed hazard assessment for 22 detected DBPs, which, although they represent only a fraction of the effluent’s composition, are insightful in identifying environmental concerns. A detailed hazard assessment is provided in Section S1 of the supporting information, and this section offers a concise summary.

The environmental fate and behavior of these 22 DBPs were analyzed using the US EPA EPI Suite [81]. DBP IDs 17 and 15 were categorized as very hydrophobic, whereas DBP IDs 9, 16, and 18 were identified as hydrophilic, indicating their bioaccumulation and bioavailability traits. Most DBPs exhibited negligible to low soil sorption, except for DBP ID 17, which showed very strong sorption. DBP ID 13 was classified as substantially volatile, and DBP ID 17 indicated moderate bioaccumulation potential.

Persistence, evaluated using BIOWIN models, classified 11 DBPs (IDs: 1, 3, 5, 10, 12, 15, 16, 17, 20, 21, 22) as potentially persistent according to REACH Guidance R.11: PBT/vPvB Assessment [82]. Toxicity assessments using ECOSAR revealed no acute toxicity among the DBPs, but DBP ID 8 exhibited the highest chronic toxicity to daphnia. The Toxicity Estimation Software Tool (TEST) version 4.1 predicted developmental toxicity in several DBPs, and mutagenicity in DBP IDs 1, 6, 16, 18, and 19. Estrogen receptor binding activity was predicted for DBP IDs 14 and 16.

Insights from the CompTox Chemicals Dashboard v2.2.1 [83] on bioassays were available only for DBP IDs 1, 2, 4, 7, 14, 15, and 16. DBP 2 underwent the highest number of tests, but exhibited activity in only 1.73% of the 984 bioassays conducted. In contrast, DBP 4 and DBP 16 showed the highest activity, with more than 50% activity observed across 235 bioassays. DBP 4 demonstrated activity in assays targeting specific receptors or pathways, such as the estrogen receptor, aryl hydrocarbon receptor, and nuclear receptors (PPARγ and PPARδ). It also exhibited activity in assays assessing enzyme inhibition or agonism (aromatase and histone deacetylase), as well as viability, apoptosis (CASP3), DNA damage (H2AX), and gene regulation (p53, ARE). DBP 16 showed activity in assays evaluating the activation or inhibition of various cellular pathways, including AhR, AP-1, AR, ARE, and CAR. It also exhibited activity in assays related to aromatase inhibition, p53 activity, nuclear receptors (ERa, ERb, ERR, FXR, GR, PPARd, PPARg, PR, PXR, RXR, VDR), and other cellular processes. These assays provide valuable information on the toxicological effects and mechanisms of the tested DBPs, shedding light on their potential biological effects and the toxicological implications of reusing the treated wastewater effluent. 

It is important to acknowledge that the analysis focused solely on the individual toxicity of the substances and did not account for mixture toxicity. Although the results provide valuable insights into the toxicity of individual substances, they do not encompass the potential additive, interactive, or synergistic effects that may arise when substances are present together in a mixture. Mixture toxicity is a complex area of study that necessitates specialized analysis and considerations. Hence, further research is necessary to assess the effects of mixtures and their overall toxicity in real-world scenarios.

## 4. Limitations and Future Outlook

This study, while yielding valuable insights regarding non-target screening of DBPs, encounters specific limitations. The peak assignment for DBPs remains provisional, as no analytical reference standards were utilized. Despite a systematic approach involving sequential filtering, isotopic pattern similarity, mass accuracy, and retention index comparison, these methods yield only tentative assignments. Accurate identification requires comparison with pure reference standards, which was not feasible in this study due to practical constraints, such as limited availability and high costs. Future research should aim to incorporate such standards for more definitive and reliable identification of DBPs.

In this study, PDMS passive samplers were utilized due to their broad-spectrum affinity for organic micropollutants. To improve both selectivity and accuracy in identifying candidates, future research should consider the simultaneous deployment of PDMS with other polymer samplers, such as POM, PE and PA. These polymers may exhibit distinct partitioning affinities for DBPs, potentially leading to more precise and accurate identification of these compounds.

The study also highlights the importance of molecular ion peak visibility in EI ionization, especially for DBPs with functional groups such as alcohols, nitrogens, carboxylic acids, esters, or extensive branching. Soft ionization techniques like chemical ionization or photoionization are recommended for more accurate detection of molecular ions in such cases.

The use of GC×GC retention indices, based on the Abraham solvation model, was a key aspect of this study. However, the use of directly measured retention indices could significantly improve the accuracy of the elution pattern analysis. Future non-target analyses should consider incorporating these directly measured indices for enhanced precision.

Furthermore, certain DBPs, due to their intrinsic properties like high polarity or thermal instability, are not suitable for gas chromatography analysis. Liquid chromatography-mass spectrometry (LC-MS) has proven to be an effective alternative, capable of handling these DBPs with higher efficacy. 

Lastly, the hazard screening of tentatively identified DBPs was performed using EPI Suite and TEST tools. To improve the precision and depth of hazard assessments, future studies could implement more sophisticated models such as those based on the Abraham Solvation Model or COSMO-RS theory, offering more detailed insights into the potential risks and environmental impact of the identified DBPs.

## 5. Conclusions

This research showcased, for the first time, the effective combination of passive sampling, GC×GC-TOFMS, NMF deconvolution, and similarity scores based on the isotopic pattern and GC×GC elution pattern for the nontarget analysis of novel and legacy DBPs. This integrated approach, particularly the synergy between passive samplers and GC×GC, proved highly effective in analyzing DBPs in wastewater. The application of the inverse watershed algorithm significantly enhanced peak picking, while NMF spectral deconvolution improved match factor scores and revealed additional DBPs. Our method successfully identified 22 potential DBPs. Further analysis based on isotopic pattern similarity showed a close correspondence between the theoretical and measured isotopologues for 11 DBPs for which molecular ion peaks were unambiguously visible in the reference spectra. The GC×GC elution patterns predicted based on the Abraham solute descriptors of detected DBPs agreed well with the observed pattern of peaks. 

The study also delved into the fate and behavior of DBPs, underscoring their sorption, volatility, and bioconcentration potential. Persistence screening identified 11 DBPs as potentially persistent, highlighting environmental degradation concerns. Toxicity assessment revealed substantial risks to aquatic organisms, with indications of developmental toxicity, mutagenicity, and endocrine disruption. Some DBPs showed activity in the CompTox bioassay database, suggesting their role in various cellular pathways.

These findings underscore the need for further research to comprehensively evaluate the environmental impacts of persistent DBPs and develop effective mitigation strategies for their presence in wastewater effluents. Additionally, understanding the potential health risks associated with DBPs remains vital for water safety and public health.

## Figures and Tables

**Table 1:** Tentative assignments of peaks to disinfection byproducts.

Number ID	Retention I (min)	Retention II (s)	Compound Name	Library CAS #	Match Factor	Reverse Match Factor	Layers
1	19.73	2.12	Benzenamine, 2,4-dichloro-	554-00-7	933	941	1
2	18.65	2.17	Benzonitrile, 2,6-dichloro-	1194-65-6	921	928	1
3	25.26	2.73	Benzenamine, 2,3,4-trichloro-	634-67-3	905	909	1
4	11.93	1.5	Acetaldehyde, tribromo-	115-17-3	893	898	1
5	21.9	2.37	2-Bromo-4-chloroaniline	873-38-1	893	911	1
6 ^a^	13.45	0.41	Bromodichloroacetaldehyde	34619-29-9	888	900	1
7	17.89	1.75	Benzaldehyde, 2,4-dichloro-	874-42-0	872	926	1
8	22.55	2.58	1H-Pyrazole, 3,4-dibromo-	5932-18-3	869	908	1
9 ^a^	11.72	1.34	Acetic acid, dibromo-, methyl ester	6482-26-4	869	904	1
10	23.85	2.17	4-Bromo-2,6-dichloroaniline	697-86-9	859	901	1
11 ^a^	26.67	3.35	Benzene, 1,1’-(bromomethylene)bis-	776-74-9	854	932	1
12	10.2	0.98	Chlorodibromoacetaldehyde	64316-11-6	851	909	1
13	10.31	0.52	Hexane, 2-bromo-	3377-86-4	841	883	2
14	16.48	1.65	Phenol, 4-chloro-	106-48-9	836	905	1
15 ^a^	26.88	2.53	Phenol, 2,4,6-tribromo-	118-79-6	834	930	2
16 ^a^	10.85	1.19	2-Propanone, 1,1,3-trichloro-	921-03-9	832	864	1
17	40.75	3.92	Acridine, 4,5-dibromo-	209460-03-7	830	872	1
18	21.36	2.73	1,1,3,3-Tetrabromoacetone	22612-89-1	821	862	1
19	16.37	1.96	Tribromoacetic acid, methyl ester	3222-05-7	821	898	1
20 ^a^	22.33	1.5	2,4,6-Trichlorophenyl isocyanate	2505-31-9	818	911	2
21	26.12	2.48	2,6-Dibromo-4-chloroaniline	874-17-9	810	864	1
22 ^a^	21.68	1.86	Benzenamine, 2,4,5-trichloro-	636-30-6	810	854	1

^a^ This peak was not detected in the raw chromatograms; instead, it was only observed in the layers obtained after the NMF deconvolution. # CAS = Chemical Abstracts Service

**Table 2:** Isotopic pattern analysis, mass accuracy, and first dimension retention index data for detected disinfection byproducts.

ID	Compound Name	CAS	Molecular Ion in Reference Data	Cosine Similarity on Isotopic Patterns	Theoretical Monoisotopic Mass (Da)	Measured Possible Monoisotopic Mass (Da)	Mass Error (ppm)	dI ^b^
1	Benzenamine, 2,4-dichloro-	554-00-7	appeared	0.998	160.9799	160.9691	67.1	7
2	Benzonitrile, 2,6-dichloro-	1194-65-6	appeared	0.998	170.9643	170.9521	71.4	9
3	Benzenamine, 2,3,4-trichloro-	634-67-3	appeared	0.997	194.9409	194.9260	76.6	NA
4	Acetaldehyde, tribromo-	115-17-3	not appeared	NA	277.7578	NA	NA	NA
5	2-Bromo-4-chloroaniline	873-38-1	appeared	0.995	204.9294	204.9137	76.7	7
6	Bromodichloroacetaldehyde	34619-29-9	not appeared	NA	189.8588	NA	NA	NA
7	Benzaldehyde, 2,4-dichloro-	874-42-0	appeared	0.75 ^a^	173.9639	173.9500	79.9	NA
8	1H-Pyrazole, 3,4-dibromo-	5932-18-3	appeared	0.999	223.8585	223.8421	73.3	NA
9	Acetic acid, dibromo-, methyl ester	6482-26-4	appeared	1	229.8578	229.8424	66.9	−19
10	4-Bromo-2,6-dichloroaniline	697-86-9	appeared	0.998	238.8904	238.8728	73.9	NA
11	Benzene, 1,1′-(bromomethylene)bis-	776-74-9	not appeared	NA	246.0044	NA	NA	25
12	Chlorodibromoacetaldehyde	64316-11-6	not appeared	NA	233.8083	NA	NA	NA
13	Hexane, 2-bromo-	3377-86-4	faint peak	NA	164.0201	NA	NA	−122
14	Phenol, 4-chloro-	106-48-9	appeared	0.999	128.0029	127.9937	72.2	−30
15 ^a^	Phenol, 2,4,6-tribromo-	118-79-6	appeared	1	327.7734	327.7469	80.8	67
16 ^a^	2-Propanone, 1,1,3-trichloro-	921-03-9	faint peak	0.995	159.9249	159.9128	75.8	2
17	Acridine, 4,5-dibromo-	209460-03-7	appeared	0.999	334.8945	334.8681	78.8	NA
18	1,1,3,3-Tetrabromoacetone	22612-89-1	faint peak	0.402	369.6839	369.6469	100.2	NA
19	Tribromoacetic acid, methyl ester	3222-05-7	faint peak	0.519	307.7683	307.7469	69.6	14
20 ^a^	2,4,6-Trichlorophenyl isocyanate	2505-31-9	appeared	0.998	220.9202	220.9028	78.6	NA
21	2,6-Dibromo-4-chloroaniline	874-17-9	appeared	1	282.8399	282.8178	78.2	NA
22 ^a^	Benzenamine, 2,4,5-trichloro-	636-30-6	appeared	0.994	194.9409	194.9271	70.7	−34

^a^ Interference of monoisotopic pattern by spectral overlap of dehydrogenated ion. ^b^ The difference between the retention index value calculated using Abraham solute descriptors and the measured retention index value reported in the NIST Chemistry WebBook is represented as dI. The term “NA” is used to denote instances where dI is not available because the measured retention index data are not available.

## Data Availability

The data presented in this study are available in Supplementary Material.

## Supplementary Materials

The following supporting information can be downloaded at: https://www.mdpi.com/article/10.3390/jox14020033/s1. Additional insights on the fate, behavior, and toxicity assessment of DBPs, including figures on DBP predictions using EPI Suite software, and tables detailing experimental and estimated Abraham solute descriptors for GC×GC elution space construction and GC×GC retention times for detected DBPs are available with this manuscript.
