# Supplementary material for: Screening Disinfection Byproducts in Arid-Coastal Wastewater: A Workflow Using GC×GC-TOFMS, Passive Sampling, and NMF Deconvolution Algorithm
Source: J Xenobiot. 2024 May 1;14(2):554–74. doi: 10.3390/jox14020033 (PMC11130967; doi:10.3390/jox14020033)
Supplement: Supplementary file 1 [file jox-14-00033-s001.zip › jox-2936841-supplementary.pdf]

Journal of Xenobiotics

MDPI

Supplementary Materials: Screening Disinfection Byproducts in Arid-Coastal Wastewater: A Workflow Using GC×GC-TOFMS, Passive Sampling, and NMF Deconvolution Algorithm

Muhammad Usman Siddiqui, Muhammad Sibtain, Farrukh Ahmad, Yasuyuki Zushi and Deedar Nabi

Contents

Fate and Behavior of Disinfection Byproducts S1

Persistence and Toxicity Assessment of Disinfection Byproducts. S3

References. S7

Figure S1: Fate and behavior of identified disinfection byproducts (DBPs) predicted using EPI Suite software.. .... S2

Figure S2: Persistence and toxicity assessment of 22 disinfection byproducts (DBPs) using EPI Suite™ modules. S3

Table S1: Available experimental values of Abraham solute descriptors used to construct GC×GC Elution Space for detected DBPs. S4

Table S2: Estimated values of Abraham solute descriptors used to construct GC×GC Elution Space for 22 detected DBPs. S5

Table S3: The GC×GC retention times (rt1, rt2), predicted first-dimension retention index data (I1) and second dimension retention parameter ($1.6^{\Delta I}$) using both experimental and estimated Abraham solute descriptors (ASDs), and the experimental first-dimension retention index (Exp I1) from the NIST Webbook. S6

Fate and Behavior of Disinfection Byproducts

Figure 6 illustrates the classification of the identified disinfection byproducts (DBPs). Based on the log K_{OC} classification provided in the EPI Suite documentation (Figure 6a), the 22 identified DBPs can be grouped as follows: One DBP very strongly sorbs to soil organic matter with negligible migration to groundwater. Two DBPs exhibit strong sorption but have slow migration to groundwater. Seven DBPs moderately sorb to soil or sediment with slow migration to groundwater. Five compounds show low sorption but moderate migration to groundwater. Lastly, seven DBPs have negligible sorption but moderate migration to groundwater. These findings indicate that DBPs with low K_{OC} values are more mobile in water, increasing the risk of reaching groundwater when untreated wastewater containing these DBPs is used for reclamation.

According to the log K_{OW} values (Figure 6b), three out of the 22 DBPs are classified as hydrophilic, indicating rapid solubility, while three DBPs are hydrophobic, suggesting a high potential for bioaccumulation. While remaining are classified as moderately bioavailable.

Analyzing the Henry's Law constant (HLC) values (Figure 6c), one out of the 22 DBPs are highly volatile from water. Additionally, three DBPs exhibit moderate volatility, while the majority of DBPs (15 out of 22) show slight volatility. Three are classified as nonvolatile. These findings indicate that some highly volatile DBPs can affect air quality.

Among the identified DBPs, none demonstrate high bioconcentration potential (Figure 6c). However, one DBP shows moderate bioconcentration potential, indicating a moderate tendency to accumulate in aquatic organisms. The majority of DBPs (21 out of 22) demonstrate very low bioconcentration potential in aquatic environments, suggesting limited accumulation in organisms.

These findings emphasize the importance of regular monitoring of the identified DBPs due to their bioaccumulative and volatile nature, which impacts their behavior and distribution in the environment. Notably, some prominent DBPs include acridine, 4,5-dibromo-; benzene, 1,1'-(bromomethylene)bis-; 2,4,6-trichlorophenyl isocyanate; and hexane, 2-bromo-. Monitoring these specific DBPs is crucial for assessing their potential impacts on human health and the environment, as well as for implementing appropriate mitigation measures to safeguard water quality and ecosystem integrity.

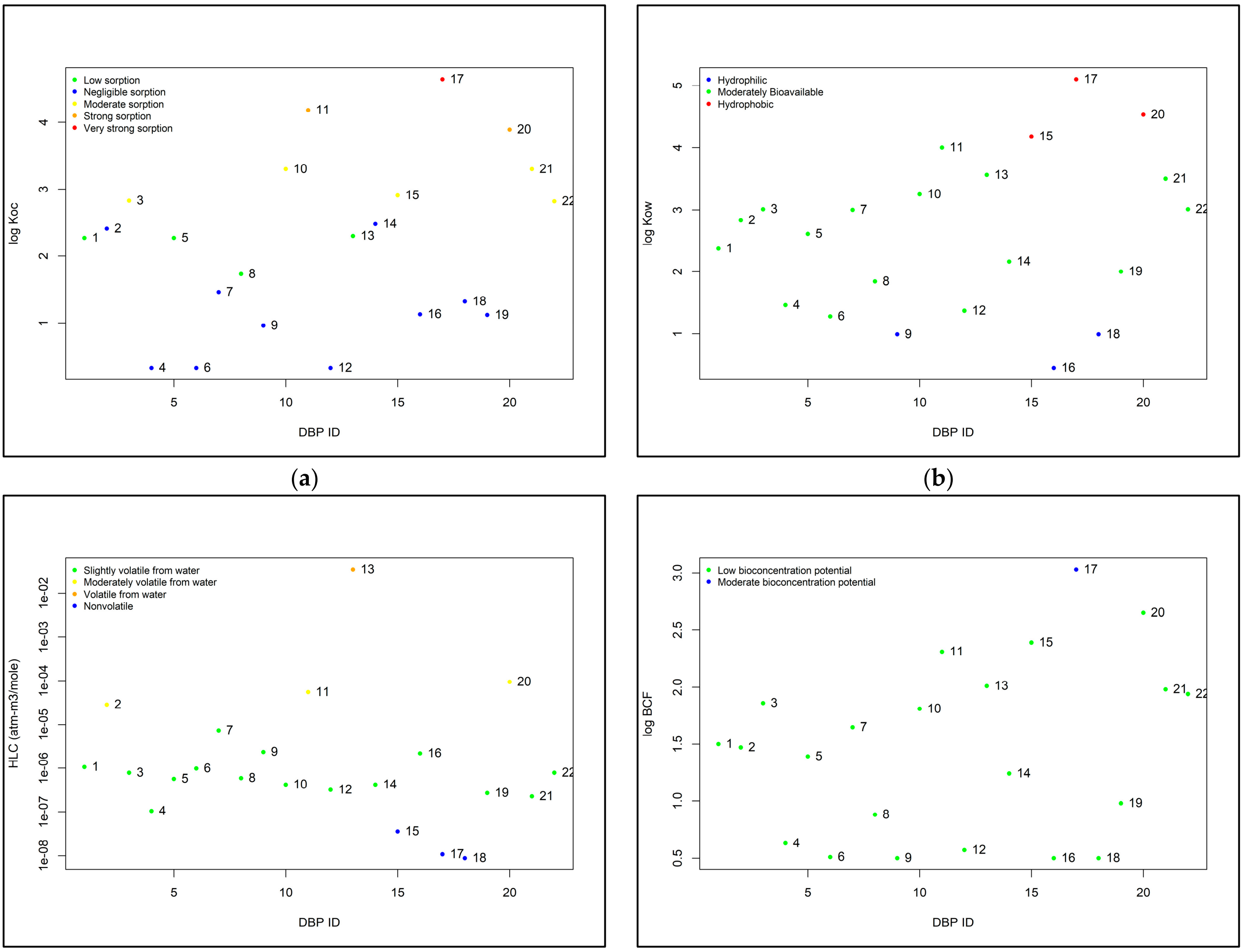

(a) (b) (c) (d)

Figure S1. Fate and behavior of identified disinfection byproducts (DBPs) predicted using EPI Suite software. (a) Soil adsorption coefficient (KOC), (b) Octanol-water partition coefficient (log KOW), (c) Henry Law constant, and (d) Bioconcentration factor (BCF) of 22 detected DBPs classified according to EPI Suite™ Manual guidelines. Data labels correspond to the serial numbers of DBPs shown in Table 1.

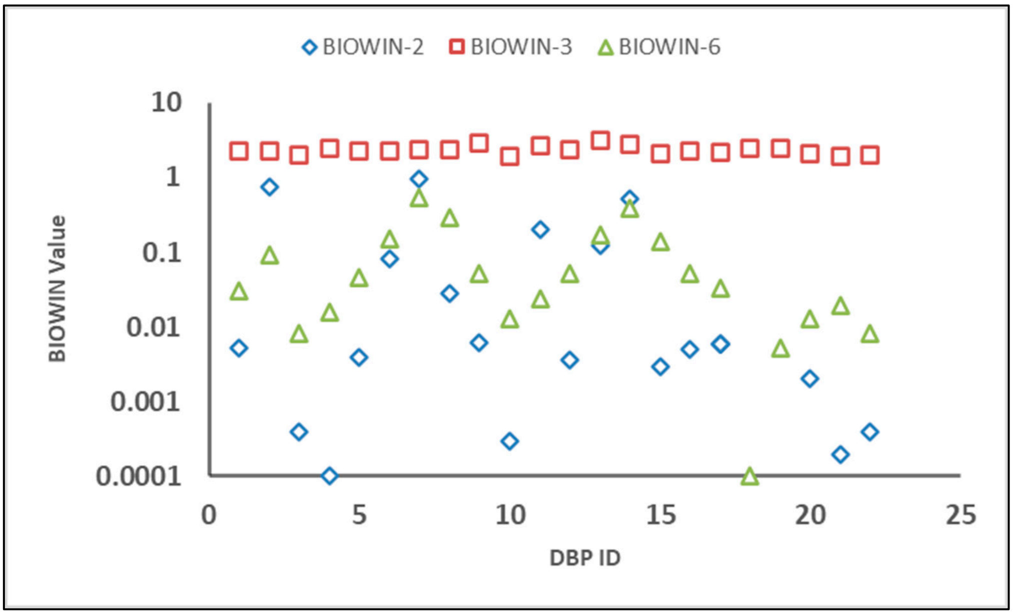

(**a**)

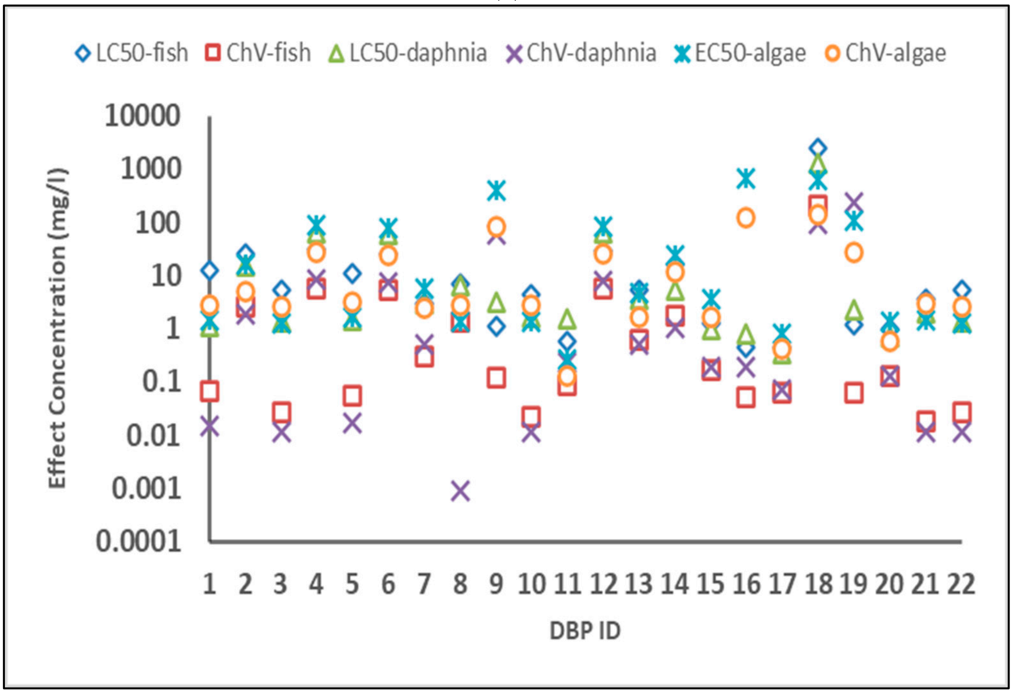

(**b**)

Figure S2. Persistence and toxicity assessment of 22 disinfection byproducts (DBPs) using EPI Suite™ modules, BIOWIN and ECOSAR, respectively. (a) BIOWIN values of DBPs obtained from BIOWIN-2, BIOWIN-3, and BIOWIN-6 models. (b) Effect concentrations obtained by ECOSAR for fish, daphnia, and algae. LC50, ChV, and EC50 represent the median lethal concentration, chronic value, and median effect concentrations, respectively. The horizontal dotted red line indicates the ECHA's criterion for acute toxicity, set at 0.01 mg/l. DBP ID corresponds to the serial numbers of DBPs shown in Table 1.

Persistence and Toxicity Assessment of Disinfection Byproducts.

The persistence of 22 DBPs was assessed using various BIOWIN models (Figure 7a). The biodegradability probability scores of 11 DBPs (DBP ID: 2, 4, 6, 7, 8, 9, 11, 13, 14, 18, 19) in the BIOWIN 3 model indicate that they are likely to undergo biodegradation within weeks or days. These DBPs have scores above 0.5, suggesting rapid biodegradability. In contrast, the remaining 11 DBPs (DBP ID: 1, 3, 5, 10, 12, 15, 16, 17, 20, 21, 22) were classified as potentially persistent according to the REACH Guidance R.11: PBT/vPvB Assessment.[1] According to this guidance, substances are considered potentially persistent if they meet either of the following criteria: BIOWIN 2 < 0.5 and BIOWIN 3 < 2.2, or BIOWIN 6 < 0.5 and BIOWIN 3 < 2.2. These DBPs may have a slower biodegradation rate or persistence in the environment.

Furthermore, the BIOWIN-7 model predicts that six DBPs (DBP ID: 7, 9, 13, 14, 18, 19) have a high probability of fast anaerobic degradation. In contrast, the remaining DBPs are not likely to undergo fast anaerobic biodegradation.

This classification allows us to differentiate between DBPs that are readily biodegradable, potentially persistent, or predicted to undergo fast anaerobic degradation, providing valuable insights into their environmental behavior and potential implications.

The toxicity evaluation in this study encompassed three trophic levels: fish, daphnia, and algae, focusing on both acute and chronic endpoints (Figure 7b). The toxicity values obtained from the ECOSAR module of EPI Suite™ provided valuable insights into the potential effects of the substances on these organisms. By including fish, daphnia, and algae in the analysis, a comprehensive assessment of the substances' impact on organisms occupying different positions in the food chain was achieved.

DBP ID 17 exhibited the highest toxicity to fish, with an LC50 value of 0.456 mg/L, highlighting its significant impact on fish populations. In contrast, DBP ID 11 demonstrated the lowest toxicity to fish, with a relatively higher LC50 value of 0.577 mg/L. When considering Daphnia, DBP ID 16 emerged as the most toxic, with the lowest LC50 value of 0.82 mg/L, suggesting a pronounced adverse effect on Daphnia populations. Conversely, DBP ID 10 displayed the least toxicity to Daphnia, with a higher LC50 value of 1.675 mg/L. Regarding algae, DBP ID 9 proved to be the least toxic, with the highest EC50 value of 405.158 mg/L. Conversely, DBP ID 11 exhibited the highest toxicity to algae, with a lowest EC50 value of 0.274 mg/L, signifying its strong inhibitory effect on algae growth.

According to the classification criterion used in this study, based on the REACH Guidance R.11: PBT/vPvB Assessment,[1] which defines a threshold of E(L)C50 < 0.1 mg/L for substances to be considered toxic, none of the DBPs met this criterion. The E(L)C50 values ranged from 0.451 to 2468.124 mg/L for LC50 in fish, from 0.012 to 243.951 mg/L for LC50 in daphnia, and from 0.274 to 659.68 mg/L for EC50 in algae. Therefore, based on these findings, it can be concluded that the examined substances did not demonstrate acute toxicity according to the specified criterion.

The potential of 22 DBs for developmental toxicity, mutagenicity, endocrine disruption was assessed using the Toxicity Estimation Software Tool (TEST) available on the CompTox Chemicals Dashboard v2.2.1.[2,3] For developmental toxicity, DB IDs 1, 6, 9, 12, 13, 16, 17, 18, and 19 were predicted to be active, indicating a potential for developmental toxicity, while the remaining substances were predicted to be inactive. In the Ames mutagenicity test, DB IDs 1, 6, 16, 18, and 19 were predicted to be active, suggesting a potential for mutagenicity, while the rest were predicted to be inactive. Estrogen receptor binding activity was predicted for DB IDs 14 and 16, while the rest of the substances were predicted to be inactive. These predictions provide valuable insights into the potential toxicity and activity of the tested substances, but further experimental validation is necessary to confirm these findings.

Table S1. Available experimental values of Abraham solute descriptors used to construct GC×GC Elution Space for detected DBPs.

DBP ID	Name	CAS-RN	SMILES	E	S	A	B	V	L	B0	Literature
1	2,4-Dichloroaniline	554-00-7	Clc1ccc(c(c1)Cl)N	1.14	1.15	0.3	0.19	1.061	5.491	0.19	Abraham Absolv
2	2,6-Dichlorobenzonitrile	1194-65-6	N#Cc1c(Cl)cccc1Cl	1.1	1.22	0	0.27	1.1159	5.573	0.27	Abraham Absolv
14	4-Chlorophenol	106-48-9	Oc1ccc(cc1)Cl	0.92	1.08	0.67	0.2	0.8975	4.775	0.2	Abraham Absolv
15	2,4,6-tribromophenol	118-79-6	Brc1cc(Br)c(c(c1)Br)O	1.62	1.34	0.5	0.16	1.3001	7.105	0.16	Abraham Absolv
22	2,4,5-Trichloroaniline	636-30-6	Clc1cc(Cl)c(cc1N)Cl	1.24	1.15	0.3	0.14	1.1834	5.927	0.14	Abraham Absolv

Source: [4]

Table S2. Estimated values of Abraham solute descriptors used to construct GC×GC Elution Space for 22 detected DBPs.

DBP ID	E_{QSPR}	S_{QSPR}	A_{QSPR}	B_{QSPR}	V	L_{QSPR}	warning $_E$	warning $_S$	warning $_A$	warning $_B$	warning $_L$
1	1.14	1.17	0.27	0.29	1.061	5.576	high leverage		out of domain		
2	1.07	1.25	0.02	0.22	1.1159	5.667					
3	1.35	1.25	0.27	0.28	1.1834	6.368	low similarity		out of domain	low similarity	
4	1.06	1.39	0	0.27	0.9311	4.447	high leverage low similarity	high leverage	no fragment overlap	low similarity	
5	1.21	1.18	0.27	0.29	1.1136	5.992	structural outlier		out of domain		
6	0.73	1.19	0	0.27	0.8259	3.616	low similarity		no fragment overlap	low similarity	low similarity
7	1.13	1.15	0	0.3	1.1178	5.561			no fragment overlap		low similarity
8	1.18	0.87	0.68	0.26	0.8863	5.343	low similarity	high leverage low similarity	structural outlier	low similarity	out of domain
9	0.75	0.95	0	0.47	0.9557	4.057			no fragment overlap	high leverage	high leverage low similarity
10	1.37	1.26	0.27	0.26	1.236	6.76	structural outlier		out of domain		
11	1.58	1.32	0	0.53	1.6401	7.234			no fragment overlap	low similarity	low similarity
12	0.89	1.29	0	0.27	0.8785	4.031	low similarity		no fragment overlap	low similarity	low similarity
13	0.32	0.25	0	0.06	1.129	3.754			no fragment overlap		
14	0.92	0.92	0.53	0.25	0.8975	4.793			low similarity		high leverage
15	1.66	1.07	0.41	0.15	1.3001	7.304			out of domain		out of domain
16	0.6	1.08	0	0.79	0.9142	3.973			no fragment overlap	leverage > 1	low similarity
17	2.82	1.3	0	0.22	1.7633	10.174	structural outlier		no fragment overlap	low similarity	
18	1.44	1.32	0	0.52	1.247	6.111	structural outlier	out of domain	no fragment overlap	out of domain structural outlier	low similarity

DBP ID	E QSPR	S QSPR	A QSPR	B QSPR	V	L QSPR	warning E	warning S	warning A	warning B	warning L
19	0.98	1.2	0	0.35	1.1307	5.148	high leverage		no fragment overlap		low similarity
20	1.21	1.32	0	0.6	1.297	6.683	high leverage		no fragment overlap		out of domain
21	1.53	1.27	0.27	0.26	1.2886	7.176	structural outlier		out of domain		
22	1.35	1.25	0.27	0.34	1.1834	6.344			out of domain		

Color codes are explained in the source: [4]

Table S3. The GC×GC retention times (rt1, rt2), predicted first-dimension retention index data (I1) and second dimension retention parameter ($1.6^{\Delta I}$) using both experimental and estimated Abraham solute descriptors (ASDs), and the experimental first-dimension retention index (Exp I1) from the NIST Webbook.

DBP ID	*rt1 (min)*	*rt2 (sec)*	*Predicted I_1 (with exp ASDs)* [a]	*Predicted $1.6^{\Delta I}$ (with exp ASDs)*	*Predicted I_1 (with est ASDs)* [a]	*Predicted $1.6^{\Delta I}$ (with est ASDs)*	*Exp I_1 (NIST Webbook)* [b]	*Comment*
1	19.73	2.12	12.76	4.003	12.93	4.057	1286	TR-1 (100% dimethyl polysiloxane)
2	18.65	2.17	12.75	4.009	12.99	4.113	1290	OV-1, SE-30, Methyl silicone, SP-2100, OV-101, DB-1, etc.
3	25.26	2.73			14.59	4.577		
4	11.93	1.5			10.61	4.666		
5	21.9	2.37			13.78	4.157	1371	TR-1 (100% dimethyl polysiloxane)
6	13.45	0.41			8.78	3.598		
7	17.89	1.75			12.67	3.777		
8	22.55	2.58			12.53	3.328		
9	11.72	1.34			9.47	2.881	966	HP-1
10	23.85	2.17			15.39	4.641		
11	26.67	3.35			16.18	4.885	1593	DB-1
12	10.2	0.98			9.70	4.093		
13	10.31	0.52			8.30	1.356	952	Kovats, Apiezon L, 125 C
14	16.48	1.65	11.56	3.830	11.35	3.208	1165	SE-30
15	26.88	2.53	16.33	5.513	16.43	4.238	1576	Ultra-1
16	10.85	1.19			9.42	3.154	940	OV-101
17	40.75	3.92			22.05	6.262		
18	21.36	2.73			13.91	4.740		
19	16.37	1.96			11.88	3.833	1174	HP-1
20	22.33	1.5			15.08	4.511		
21	26.12	2.48			16.23	4.849		
22	21.68	1.86	13.64	4.090	14.54	4.577	1488	TR-1 (100% dimethyl polysiloxane

[a] multiply values in this column by 100 to compare them with experimental retention index data given in column, Exp I_1 (NIST Webbook). [b] Exp I_1 (NIST Webbook) indicate the experimental retention index value available in NIST Webbook [5].

References

1. ECHA Guidance on Information Requirements and Chemical Safety Assessment - Chapter R.11: PBT/VPvB Assessment. *Eur. Chem. Agency* **2017**.
2. US EPA CompTox Chemicals Dashboard Available online: https://comptox.epa.gov/dashboard (accessed on 1 May 2023).
3. Williams, A.J.; Grulke, C.M.; Edwards, J.; McEachran, A.D.; Mansouri, K.; Baker, N.C.; Patlewicz, G.; Shah, I.; Wambaugh, J.F.; Judson, R.S.; et al. The CompTox Chemistry Dashboard: A Community Data Resource for Environmental Chemistry. *J. Cheminform.* **2017**, *9*, doi:10.1186/s13321-017-0247-6.
4. Ulrich, N.; Endo, S.; Brown, T.N.; Watanabe, N.; Bronner, G.; Abraham, M.H.; Goss, K.-U. UFZ-LSER Database v 3.2.1 Available online: http://www.ufz.de/lserd. (accessed on 11.11.2023)
5. Wallace, W.E. NIST Chemistry WebBook, SRD 69, Retention Indices" by NIST Mass Spectrometry Data Center Available online: https://webbook.nist.gov/chemistry (accessed on 01. 11. 2023).
